# 3′-Phosphoadenosine 5′-phosphosulfate synthase 1 (PAPSS1) knockdown sensitizes non-small cell lung cancer cells to DNA damaging agents

**DOI:** 10.18632/oncotarget.3635

**Published:** 2015-04-11

**Authors:** Ada W. Y. Leung, Wieslawa H. Dragowska, Daniel Ricaurte, Brian Kwok, Veena Mathew, Jeroen Roosendaal, Amith Ahluwalia, Corinna Warburton, Janessa J. Laskin, Peter C. Stirling, Mohammed A. Qadir, Marcel B. Bally

**Affiliations:** ^1^ Experimental Therapeutics, BC Cancer Research Centre, Vancouver, BC, V5Z 1L3, Canada; ^2^ Department of Pathology and Laboratory Medicine, University of British Columbia, Vancouver, BC, V6T 2B5, Canada; ^3^ Terry Fox Laboratory, BC Cancer Research Centre, Vancouver, BC, V5Z 1L3, Canada; ^4^ Department of Pharmaceutical Sciences, Utrecht University, Utrecht, TB, 3508, The Netherlands; ^5^ Medical Oncology, BC Cancer Research Centre, Vancouver, BC, V5Z1L3, Canada; ^6^ Department of Medicine, University of British Columbia, Vancouver, BC, V5Z 1M9, Canada; ^7^ Department of Medical Genetics, University of British Columbia, Vancouver, BC V6H 3N1, Canada; ^8^ Faculty of Pharmaceutical Sciences, University of British Columbia, Vancouver, BC, V6T 1Z3, Canada; ^9^ Centre for Drug Research and Development, Vancouver, BC, V6T 1Z3, Canada

**Keywords:** PAPSS1, sensitization, non-small cell lung cancer, siRNA screen, DNA damage

## Abstract

Standard treatment for advanced non-small cell lung cancer (NSCLC) with no known driver mutation is platinum-based chemotherapy, which has a response rate of only 30–33%. Through an siRNA screen, 3′-phosphoadenosine 5′-phosphosulfate (PAPS) synthase 1 (PAPSS1), an enzyme that synthesizes the biologically active form of sulfate PAPS, was identified as a novel platinum-sensitizing target in NSCLC cells. PAPSS1 knockdown in combination with low-dose (IC_10_) cisplatin reduces clonogenicity of NSCLC cells by 98.7% (*p* < 0.001), increases DNA damage, and induces G1/S phase cell cycle arrest and apoptosis. PAPSS1 silencing also sensitized NSCLC cells to other DNA crosslinking agents, radiation, and topoisomerase I inhibitors, but not topoisomerase II inhibitors. Chemo-sensitization was not observed in normal epithelial cells. Knocking out the PAPSS1 homolog did not sensitize yeast to cisplatin, suggesting that sulfate bioavailability for amino acid synthesis is not the cause of sensitization to DNA damaging agents. Rather, sensitization may be due to sulfation reactions involved in blocking the action of DNA damaging agents, facilitating DNA repair, promoting cancer cell survival under therapeutic stress or reducing the bioavailability of DNA damaging agents. Our study demonstrates for the first time that PAPSS1 could be targeted to improve the activity of multiple anticancer agents used to treat NSCLC.

## INTRODUCTION

With a 5-year survival rate of 16%, lung cancer continues to be the leading cause of cancer-related deaths [1, 2]. Although lung cancer is primarily caused by smoking, approximately 25% of worldwide sufferers never smoked (lifetime exposure of < 100 cigarettes) and there appears to be a rise in the incidence of non-smoking related lung cancers worldwide [3]. Nearly 85% of all lung cancers are attributed to non-small cell lung cancer (NSCLC), of which 65–80% patients are diagnosed with an inoperable, locally advanced or metastatic disease [4, 5]. Platinum-based combination chemotherapy, consisting of carboplatin or cisplatin in combination with a second drug such as pemetrexed, paclitaxel, or vinorelbine, has been the standard treatment for advanced NSCLC patients for the past two decades [4, 6]. In recent years, movement towards personalized medicine resulted in the development and use of EGFR, ALK, and other tyrosine kinase-targeting inhibitors as first-line treatments for patients whose tumors harbor these known driver mutations. This treatment strategy is associated with more favorable toxicity profiles and improved progression-free survival over standard chemotherapy alone but it has been difficult to demonstrate an overall survival advantage, and resistance to TKIs has been noted [7, 8]. Furthermore, only a small population of NSCLC patients in western countries has these mutations and hence, most patients still rely on platinum-based treatments in the first line setting. [9].

Based on the clinical data from various NSCLC-focused clinical trials, it is certain that cisplatin is unlikely to be replaced in the first line setting: at least for majority of patients [10]. We argue here that one of many reasons for the lack of improvement in treating NSCLC concerns the fact that new drugs are not developed in the context of cisplatin use in a first line setting. For this reason, our laboratory embarked on a siRNA screen in combination with low-dose cisplatin (IC_10_) in an attempt to identify targets that potentiate the therapeutic effects of cisplatin when the cells are first exposed to the drug. One of the premises behind the design of this screen was the belief that chemotherapy-naïve lung cancer cells that are not exposed to lethal cisplatin concentrations *in vivo* will develop cytoprotective responses. If such cytoprotective responses occur, then it will be possible to develop strategies designed to inhibit these responses. This, in turn, will be expected to increase the potency of cisplatin when first used to treat chemo-naïve NSCLC patients. A second premise concerns the potential for the screen to identify synthetic-sick interactions where an ineffective dose of cisplatin could prove very effective when added to a cell population where selected genes have been silenced. Here, we report on validation studies completed on a top hit identified in this screen. Our results demonstrate, for the first time, that silencing of 3′-phosphoadenosine 5′-phosphosulfate (PAPS) synthase 1 (PAPSS1), a bi-functional enzyme that synthesizes the universal sulfate donor PAPS [11], can enhance cisplatin activity in NSCLC cell lines by inducing apoptosis and G1/S phase cell cycle arrest. Importantly, PAPSS1 silencing also enhances the activity of radiation, other platinum agents, topoisomerase I inhibitors, but not topoisomerase II inhibitors or microtubule-targeted drugs.

## RESULTS

### siRNA screens identified PAPSS1 as a target improving cisplatin activity when silenced

**Figure 1:**
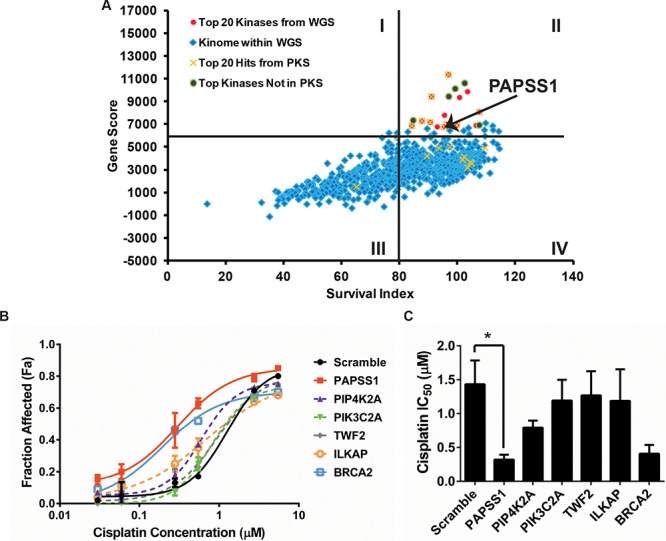
siRNA kinome screens identified PAPSS1 as a target that can be silenced to improve cisplatin activity The results from the kinome screen are summarized in **A.** where each data point represents one gene being silenced in the presence or absence of cisplatin. The x-axis indicates cell viability under gene-silencing condition in the absence of cisplatin. The Gene Score on the y-axis is calculated as the product of survival index (cell viability from gene knockdown alone) and potentiation effects (the difference in cell count in the absence versus the presence of cisplatin when the gene is silenced). Cisplatin dose response curves (72 h treatment) were generated to further evaluate the top five targets **B.** The data is plotted as fraction affected (mean ± SD) where a Fa value of 0 would indicate equivalent viable cell count in the treated well relative untreated controls. The IC_50_ values interpolated from these fitted curves (mean ± 95% confidence intervals) are displayed in C. PAPSS1 silencing caused the most reduction in IC_50_ relative to scramble (*p < 0.05) and improved cisplatin response to an equal or a greater extent compared to BRCA2 silencing.

A Preliminary Kinome Screen (PKS) comprising 640 kinases was performed prior to the Whole Genome Screen (WGS) to establish all screening parameters. Cisplatin-potentiating candidates were identified using two selection criteria: 1) gene knockdown must have little or no impact on viable cell count in the absence of cisplatin and 2) a significant decrease in cell viability must be observed in the presence of low-dose cisplatin. The lethality of the knockdown termed “survival index” here, is determined based on cell counts relative to the negative controls within the same plate: a survival index of 100% suggests that gene knockdown has no effect on cell viability. The extent of potentiation is determined by the difference in cell count in the absence versus the presence of cisplatin (IC_10_), normalized to the BRCA2 positive control. The two parameters were combined to calculate a “gene score” to rank all genes. Genes with a high “gene score” and a high survival index (quadrant II, Figure 1A) would satisfy the selection criteria as cisplatin activity enhancers. Since the WGS provided a biological replicate of the PKS, the two kinase datasets were analyzed independently to evaluate the reproducibility of our siRNA screen. The results are summarized in Figure 1 where each data point represents the results from one gene. The top 20 kinases from the PKS and WGS are highlighted in yellow crosses and red circles respectively. An overlap of 9 kinases in the two top-20 lists was observed (Figure 1A - red circles marked with X; Table S1). Five of the top 20 kinases in WGS were not part of the PKS (green circles) as the WGS had 778 kinases in total. Using the same screening parameters, the 20 kinases with the strongest potentiation effects from the PKS were re-screened three times with a pool of three siRNA duplexes (Stealth siRNA) targeting each gene which were different than those used for the WGS and PKS. The Stealth siRNAs used were also chemically modified to increase the specificity and stability of the siRNAs. Here, PAPSS1 ranked consistently in all three independent experiments, as the top cisplatin-potentiating candidate (Table S2). The sensitization observed was further confirmed by repeating the screen using the three siRNA duplexes separately to ensure that the phenotype observed is not due to off-target effects (Figure S1). Referring back to Figure 1A, PAPSS1 ranked as the 7th and 18th kinase in the PKS and WGS respectively in contrast to its other isoform, PAPSS2, which ranked at ~11, 500 of 21, 121 genes. When five of the top targets from the validation screen were further evaluated by generating cisplatin dose response curves, PAPSS1 silencing demonstrated the most leftward shift in the dose response relative to the negative control scramble siRNA (Figure 1B). This was also reflected in the IC_50_ values for cisplatin (Figure 1C). PAPSS1 inhibition when used in combination with cisplatin appeared to sensitize A549 cells to an equal or greater extent compared to BRCA2 silencing (Figure 1B–1C).

**Figure 2:**
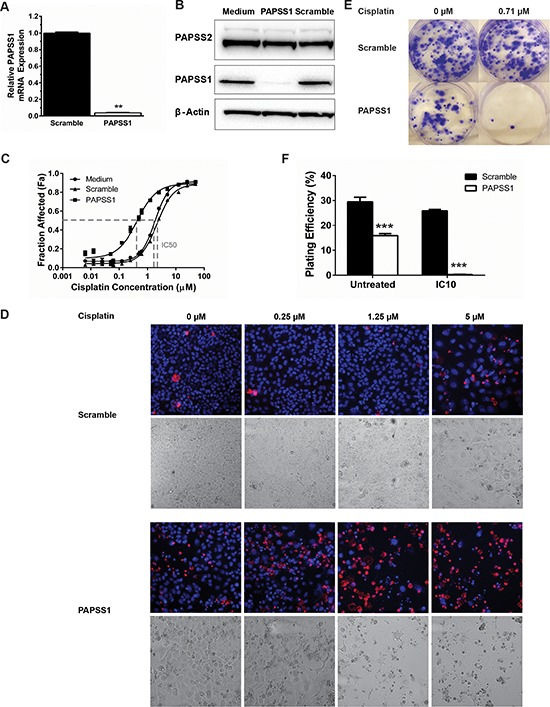
Validation of siRNA screen in A549 cells Using a pool of 3 PAPSS1-targeting siRNA duplexes, PAPSS1 expression was reduced by > 90% at the mRNA level (**A.** ^**^p < 0.01). This knockdown was further confirmed by Western blot analysis **B.** PAPSS1 silencing (solid squares) yielded a leftward shift in the cisplatin dose response curve relative to controls **C.** The representative fluorescent (upper panels) and bright field (lower panels) images at selected doses of cisplatin in cells transfected with scramble or PAPSS1 siRNA obtained with IN Cell Analyzer are shown (**D.** ;10x magnification). Cell viability was assessed based on detection of plasma membrane integrity 72 hours following cisplatin treatment. Total and dead cell counts are determined using Hoechst 33342 (blue) and ethidium homodimer (red) staining. The long-term effects of PAPSS1 knockdown with and without cisplatin were explored using clonogenic assays. A representative image of each transfection and treatment condition is shown **E.** The plating efficiency, defined as the number of colonies formed from the number of trypan blue excluding cells is plotted as means ± SEM (**F.** ***p < 0.001).

Further validation studies associating PAPSS1 silencing with enhanced cisplatin activity in A549 cells are summarized in Figure 2. Under conditions where siRNAs strongly suppressed PAPSS1 mRNA and protein levels (Figure 2A–2B), a significant shift in the cisplatin dose response curve was observed (Figure 2C). Note that the loss of PAPSS1 expression did not affect PAPSS2 expression (Figure 2B). PAPSS1 knockdown led to a 5.4-fold and 6.8-fold decrease in the cisplatin IC_50_ relative to untransfected and scramble controls, respectively. The potent combinatorial effects of PAPSS1 inhibition and cisplatin are apparent in representative images shown in Figure 2D. Although PAPSS1 silencing had little impact on cell viability in the absence of cisplatin, the images (Figure 2D) suggested that silencing alone engendered changes in cell morphology. To explore this further, the long-term effects of PAPSS1 inhibition were investigated using a clonogenic assay. As shown in the representative images in Figure 2E supported by a quantitative assessment of plating efficiency (Figure 2F), PAPSS1 knockdown alone significantly decreased the clonogenicity of A549 cells when compared to cells transfected with the scramble siRNA. More importantly, PAPSS1 inhibition in combination with low-dose cisplatin (IC_10_) reduced the plating efficiency by about 98.7% relative to scramble controls (*p* < 0.001; Figure 2E and 2F).

### PAPSS1 silencing potentiates cisplatin activity in a dose-dependent manner

**Figure 3:**
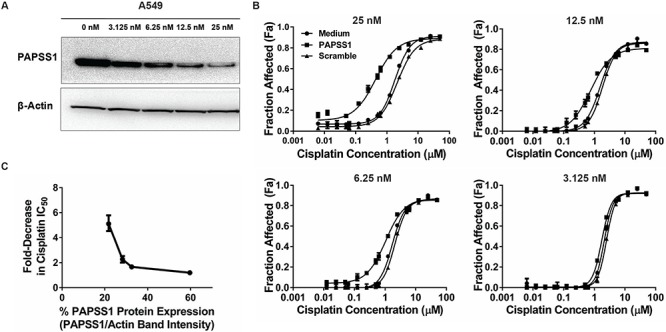
Cisplatin potentiation is dependent on the level of PAPSS1 silencing Western blot showing the siRNA dose-dependent reduction in PAPSS1 protein expression **A.** The correlation between the extent of cisplatin activity enhancement and siRNA concentration is demonstrated in the differing levels of leftward shift in the cisplatin dose response curve (**B.** ;mean ± SEM). The fold-change in the cisplatin IC_50_ was plotted against the band intensity from the western blot (**C.** ;error bars represent 95% confidence intervals). PAPSS1 expression was determined by normalizing PAPSS1 band intensity to β-Actin band intensity. The calculated value for each dose was then normalized such that 100% would be equivalent to untransfected (0 μM) control.

To assess how the level of PAPSS1 inhibition influenced cisplatin activity, A549 cells were transfected with increasing concentrations of PAPSS1 siRNA and PAPSS1 protein expression was determined by Western blot analysis (Figure 3). As shown in Figure 3A, there was an siRNA dose-dependent decrease in PAPSS1 protein levels. Next, cisplatin dose response curves (DRC) were performed in A549 cells exposed to decreasing amounts of PAPSS1 siRNA (Figure 3B). At the highest siRNA dose used (25nM), the greatest shift in the cisplatin dose response curve was observed with no significant change in the cisplatin DRC at 3.125 nM of siRNA. A densitometry-based plot of fold-decrease in cisplatin IC_50_ versus protein expression level demonstrates a correlation between reduction in PAPSS1 protein level and increases in cisplatin activity (Figure 3C); increases in cisplatin activity were greatest when PAPSS1 silencing was > 75%.

### PAPSS1 silencing potentiates cisplatin activity in NSCLC cell lines with different genetic backgrounds but does not increase cisplatin cytotoxicity in normal lung epithelial cells

**Figure 4:**
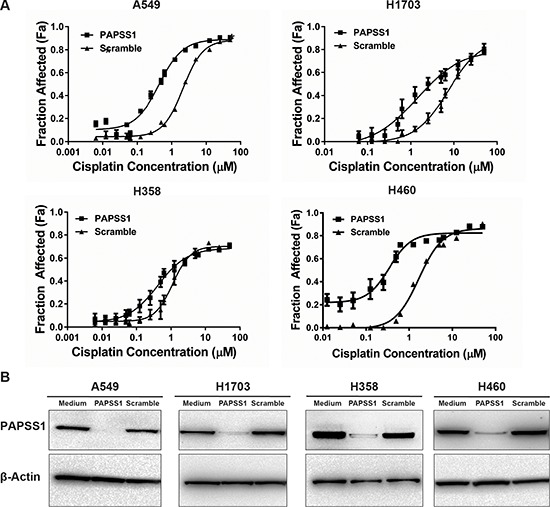
PAPSS1 silencing enhances cisplatin activity in NSCLC cell lines with different genetic background PAPSS1 was silenced using siRNA methods in a number of different non-small cell lung cancer cell lines **A.** Cisplatin cytotoxicity in scramble or PAPSS1 transfected cells **B.** ;data are plotted as mean ± SEM, representative of at least three independent experiments; *p < 0.05 relative to scramble control.

To exclude a possibility that enhancement of cisplatin activity with PAPSS1 silencing observed in A549 cells is a cell line specific event, cytotoxicity curves were produced using H358, H1703, and H460 NSCLC cells. These cell lines, including A549, all harbor wild-type EGFR but differ in their tumor subtype and p53 and KRAS mutational status (Table S3) [12]. The cytotoxicity data show that PAPSS1 silencing results in 1.8, 3.3, and 6.5-fold decrease in IC_50_ in H358, H1703, and H460 cells, respectively (Figure 4A) when significant PAPSS1 silencing was achieved at the protein level (Figure 4B). Although H460 was most sensitized to cisplatin treatment when PAPSS1 expression was inhibited, it was also most sensitive to PAPSS1 knockdown, with approximately 40% loss in cell viability within 96 hours in the absence of cisplatin (Figure S2). Since PAPSS1 silencing appeared to be less lethal in A549 cells while still engendering a 5-fold enhancement in cisplatin activity, A549 was chosen for further studies.

**Figure 5:**
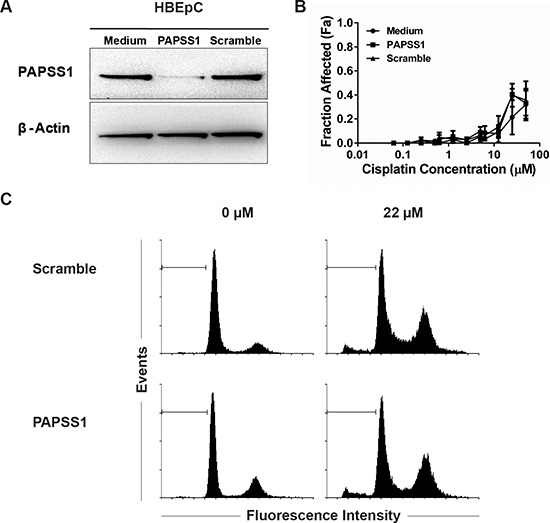
PAPSS1 silencing does not enhance cisplatin activity in HBEpC normal airway cells Gene knockdown was confirmed by Western blot analysis at 72 hours post-transfection **A.** Data from three cisplatin dose response curves were averaged and plotted as mean ± SEM **B.** HBEpC cells were also subjected to cell cycle analysis by flow cytometry **C.** ;apoptotic populations are marked with a horizontal marker.

To investigate how PAPSS1 silencing affects normal lung cells, human lung microvascular endothelial cells (HLMVEC) and human bronchial epithelial (HBEp) cells were grown to complete confluence to model non-proliferating normal tissue and then transfected with PAPSS1-targeting siRNA (or scramble control) followed by addition of cisplatin 24 hours later. In HLMVEC, substantial PAPSS1 knockdown could not be achieved (< 70% reduction at the messenger RNA level and little PAPSS1 loss at the protein level) even when using the highest siRNA concentrations possible (Figure S3). Consistent with the data in Figure 3, the PAPSS1 suppression in this primary cell line was not sufficient to determine a change in the cisplatin dose response curve. In contrast, in HBEp cells, substantial knockdown of PAPSS1 was achieved at the protein level (Figure 5A) with > 90% reduction in mRNA expression (data not shown). Over the period of 72 hours following transfection, viability of confluent HBEp cells did not decrease. Importantly, there was no significant difference in cisplatin activity observed in PAPSS1-silenced cells (Figure 5B). Similar cisplatin-induced cytotoxicity was observed in control and PAPSS1-silenced HBEp cells at high (>10 μM) cisplatin concentrations. HBEp cells transfected with PAPSS1 or scramble siRNA were also subjected to cell cycle analysis following treatment with an effective dose of cisplatin (22 μM) for 72 hours. As shown in Figure 5C, unlike the NSCLC line (see below), PAPSS1 silencing did not affect cell cycle distribution or the fraction of apoptotic cells in the normal epithelial cell population.

### PAPSS1-silencing in combination with cisplatin increases DNA damage and induces G1/S phase arrest and apoptosis

**Figure 6:**
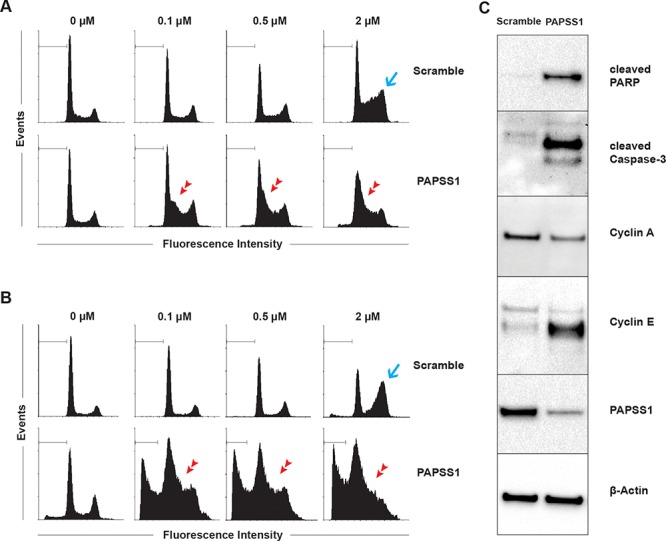
PAPSS1 knockdown in combination with cisplatin induces G1/S-phase arrest and produces high rates of apoptosis Cells were treated with selected concentrations of cisplatin for 24 **A.** or 48 hours **B.** 24 hours following transfection and the effects of both PAPSS1 silencing and cisplatin treatment on cell cycle distribution are summarized here. The apoptotic population is marked with a horizontal line. The blue arrow points to the G2/M phase block in the cell cycle while the red arrow heads indicate a G1/S phase block. Western blot analyses of the expression of cyclins and common apoptotic markers (cleaved PARP and cleaved caspase-3) are shown **C.** in A549 cells that were transfected with scramble or PAPSS1 siRNA and then treated with 0.71 μM (IC_10_) of cisplatin for 24 hours.

To gain a better understanding of the mechanism by which PAPSS1 sensitizes A549 cells to cisplatin treatment, the effects of gene knockdown on cell cycle and the levels of sub G0/G1 population, considered apoptotic, were measured by flow cytometry at 24 (Figure 6A) and 48 hours (Figure 6B) following treatment. Quantification of the cell cycle distribution and apoptotic fraction can be found in Figure S4. The data demonstrate that at both 24 and 48 hours, a G2/M block (blue arrows) was observed at the highest cisplatin dose tested in the scramble control while a marked G1/S block (red arrowheads) was observed in PAPSS1-silenced cells treated with cisplatin (Figure 6A and 6B). In addition, in the presence of cisplatin, a small increase in the population of sub G0/G1 apoptotic cells is noticeable at 24 hours in PAPSS1-silenced cells compared to scramble controls (Figure 6A) and this difference becomes very prominent at 48 hours post-treatment (Figure 6B). Consistent with the flow cytometric data, Western blot analysis shows that PAPSS1 silencing in the presence of cisplatin results in increased expression of the common apoptotic markers cleaved caspase-3 and cleaved PARP (Figure 6C). Consistent with the accumulation of cells in the G1/S phase, there is also a significant up-regulation of cyclin E and down-regulation of cyclin A expression when PAPSS1 knockdown was combined with cisplatin (Figure 6C).

**Figure 7:**
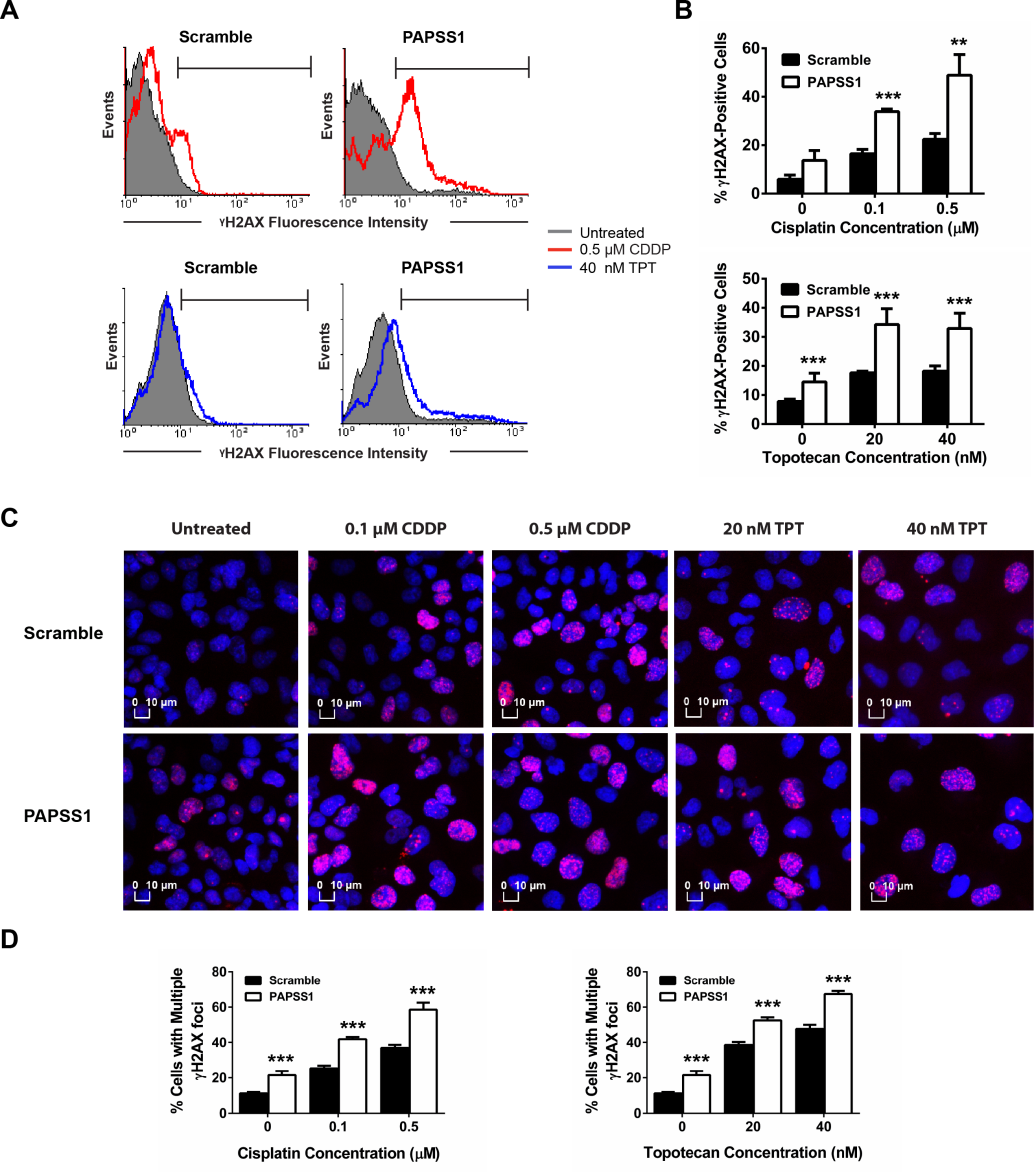
Relative to non-silenced controls, more DNA damage occurs when PAPSS1-silenced A549 cells are treated with cisplatin or topotecan The representative histograms illustrating the expression of γH2AX are shown **A.** Topotecan (250 nM for 24 hours) was used as a positive control to set up gating parameters. The quantified γH2AX population at each dose of cisplatin (CDDP) and topotecan (TPT) under PAPSS1-silencing and non-silencing conditions are plotted **B.** For immunofluorescent staining, cells were treated with CDDP or TPT, stained with anti-γH2AX antibody (red) and counterstained with Hoechst 33342 (blue) **C.** Images were visualized using the IN Cell Analyzer 2200 (20X/0.45 objective). Percentage of cells with more than one γH2AX-positive puncta are quantified and plotted in D. All data are displayed as mean ± SD; **p < 0.01, ***p < 0.001.

Based on these data, we hypothesized that PAPSS1 activity may normally be involved in mitigating cisplatin induced DNA damage or in promoting DNA repair following cisplatin treatment. To date, the most sensitive biomarker used to assess the DNA damage that correlates with DNA strand-breaks is phosphorylation of histone H2AX at Ser 139 (γH2AX) [13]. Thus, flow cytometry was performed to measure the levels of γH2AX under PAPSS1-silencing conditions (Figure 7A–7B). As shown in Figure 7A, low-dose cisplatin caused a slight increase in γH2AX in scramble controls. However, when PAPSS1 was silenced, a marked increase in γH2AX was observed. The levels of γH2AX at each tested dose are shown in the corresponding plots (Figure 7B). PAPSS1-silencing resulted in significantly more γH2AX-labeling in the presence of low doses of cisplatin (0.1 and 0.5 μM). Since topotecan, a commonly used positive control for γH2AX, is also a DNA damaging agent, we used low doses of topotecan (20 and 40 nM) to see whether the increase in γH2AX levels could be observed with a non-platinum DNA damaging agent. As shown in Figure 7A–7B, PAPSS1 knockdown in combination with low doses of topotecan resulted in greater accumulation of γH2AX. Interestingly, PAPSS1 knockdown alone appears to induce DNA damage, albeit at low levels, in both cisplatin and topotecan treated cells. These results were further confirmed using immunofluorescence staining for γH2AX and high content analysis (HCA) (Figure 7C–7D). Figure 7C shows the representative images of γH2AX foci in scramble and PAPSS1 siRNA-transfected cells treated with low doses of cisplatin or topotecan. At each dose, significantly more cells with γH2AX-positive puncta were detected in PAPSS1 silenced cells. Further, PAPSS1-silenced cells had more cells with multiple γH2AX foci, which are less likely to survive (Figure 7D). These data were consistent with the flow analysis shown in Figure 7A and 7B confirming that γH2AX expression is enhanced when PAPSS1-silenced cells are treated with low doses of cisplatin or topotecan. These data suggest that PAPSS1, when expressed at normal levels, is involved in reducing the amount of DNA damage caused by cisplatin and topotecan, either by blocking drug action or promoting efficient DNA repair.

### *MET3* and *MET14* deletion do not sensitize yeast to cisplatin treatment

In an attempt to gain a better understanding of how PAPSS1 silencing affects increases in cisplatin activity, we initiated studies in *Saccharomyces cerevisiae*. The ATP sulfurylase and APS kinase domains that make up PAPSS enzymes are encoded by two separate genes in *Saccharomyces cerevisiae*, *met3* and *met14* respectively. In yeast, the metabolite PAPS plays a role in amino acid metabolism but unlike humans, yeast do not use sulfotransferases to detoxify chemicals nor do they modify proteins by sulfation. We found that single and double deletion mutants of the yeast *MET3* and *MET14* genes were not more sensitive to cisplatin than an isogenic wildtype strain (see Figure S5). These results suggest that the phenotype observed with PAPSS1 inhibition and low-dose cisplatin in cell lines stems from a PAPSS function unique to those cells. Increases in cisplatin activity in PAPSS1-silenced cells likely involve post-translational sulfation of selected proteins.

### PAPSS1 silencing sensitizes NSCLC cells to radiation and treatments with DNA crosslinkers and topoisomerase I inhibitors

**Figure 8:**
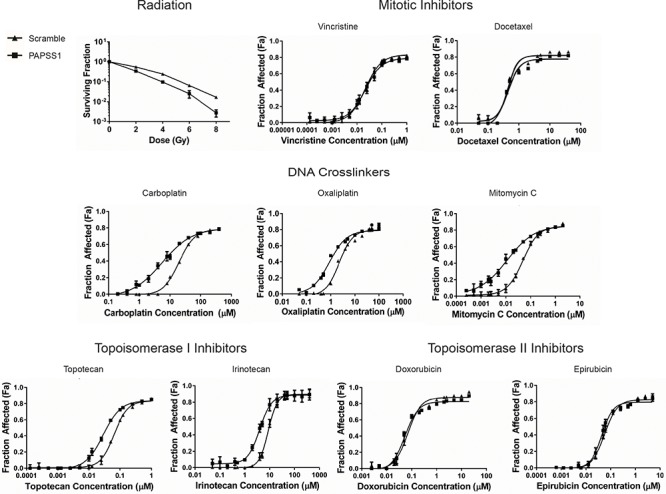
PAPSS1 silencing sensitizes A549 cells to radiation, as well as platinum-based agents and topoisomerase I inhibitors Cells transfected with PAPSS1 or scramble siRNA were subjected to selected doses of radiation. Data were normalized to non-irradiated controls (mean ± SD; *n* = 3). The dose response curves from chemotherapeutic treatments are displayed as mean ± SEM and are representative of at least three independent experiments.

Thus far the results have focused on cisplatin and we have clearly shown that in PAPSS1-silenced cells, low doses of cisplatin increase the number of cells arresting in the G1/S phase (Figure 6) and increase γH2AX-labeling (Figure 7): effects that are associated with a significant increase in cisplatin cytotoxicity even at low doses of cisplatin. Since PAPSS1 knockdown also appears to increase DNA damage in cells treated with topotecan, we speculated that the mechanisms linked to PAPSS1 silencing-induced increases in cisplatin activity are not cisplatin-specific. To test this hypothesis, we evaluated how PAPSS1-silencing would influence the activity of other DNA damaging agents. The data presented in Figure 8 show that PAPSS1 knockdown sensitized A549 cells to radiation (*p* < 0.01), reducing the surviving fraction by up to 7-fold at 8 Gy relative to scramble controls. PAPSS1 silencing also enhanced the activity of other DNA crosslinkers, such as carboplatin, oxaliplatin, mitomycin C, as well as the selected topoisomerase I inhibitors, causing at least a 2-fold decrease in the IC_50_ of these agents (Table 1). On the other hand, no changes in cytotoxicity were observed when A549 cells were treated with the selected mitotic and topoisomerase II inhibitors. These results indicate conclusively that the mechanism of PAPSS1-silencing induced enhancement of cisplatin activity is not cisplatin-specific and therefore is not directly related to the role of sulfotransferases in cisplatin metabolism. Further, the results indicate that targeting PAPSS1 has the potential to potentiate many broad spectrum genotoxic therapies; very likely through mechanisms that involve increased DNA damage or reductions in DNA repair.

**Table 1:** IC_50_ Values of Chemotherapeutics in A549 Cells

Chemotherapeutic Agent	Scramble	PAPSS1 Knockdown	*p*-value
**Mitotic Inhitibors**			
Vincristine	29.0 nM	32.0 nM	ns
Docetaxel	0.54 μM	0.61 μM	ns
**DNA Crosslinkers**			
Cisplatin	2.44 μM	0.44 μM	*p* < 0.05
Carboplatin	30.0 μM	10.2 μM	*p* < 0.05
Oxaliplatin	3.31 μM	1.41 μM	*p* < 0.05
Mitomycin C	68.2 nM	16.5 nM	*p* < 0.05
**Topoisomerase I Inhibitors**			
Topotecan	78.9 nM	37.8 nM	*p* < 0.05
Irinotecan	9.7 μM	4.3 μM	*p* < 0.05
**Topoisomerase II Inhibitors**			
Doxorubicin	83.5 nM	71.9 nM	ns
Epirubicin	69.0 nM	51.9 nM	ns

ns = IC_50_ values between control and PAPSS1-silenced cells are not statistically significant

## DISCUSSION

The typical path of developing combinations for the treatment of any cancer has been combining new drugs with “standard” drugs that are known to provide some clinical benefits. For NSCLC patients, this defines a 30-year history of clinical trials combining cisplatin with the next best drug with no meaningful gain in overall survival [14, 15]. Alternatively, approaches designed to enhance cisplatin activity in first-line therapy may provide therapeutic benefits to this patient population. Here, we applied the siRNA screening approach to identify gene targets that would enhance the cytotoxic effects of low-dose (IC_10_) cisplatin in chemo-naïve NSCLC cells. Utilizing a sub-lethal dose of cisplatin considers the fact that in solid NSCLC tumors, cells are exposed to a range of cisplatin concentrations depending on tumor architecture and microenvironment. Cancer cells exposed to high (cytotoxic) drug levels will die whereas those exposed to sub-lethal drug doses develop cytoprotective responses that promote survival. We demonstrated here, for the first time, that PAPSS1 silencing can enhance cisplatin activity in multiple NSCLC cell lines. It is important to note that silencing of PIP4K2A, the top kinase from both the PKS and WGS which has also been recently identified as a pharmaceutical target in p53-mutated cancers [16], yielded only a modest shift in the cisplatin dose response curve compared to PAPSS1 in A549 cells (Figure 1B–1C). The increase in cisplatin activity seen when PAPSS1 was silenced was comparable to that observed when BRCA2 was silenced. Although it is difficult to translate the changes observed *in vitro* to a clinical setting, BRCA2 deficiency is known to be associated with better treatment response to platinum drugs in patients [17–20].

Regarding the clinical relevance of PAPSS1 it is important to recognize that this protein is expressed in normal and cancer cells alike and based on the data shown in Figure 3 (3C), we can conclude that sensitization to cisplatin would only be observed in cell population where PAPSS1 expression is very low. At this time, there is a lack of gene expression data derived from lung cancer patients which would suggest that PAPSS1 levels are important in terms of predicting response to cisplatin-containing cocktails. We are also not aware of any database that would provide these data in the context of cisplatin treatment response. There are many research teams that are doing genomic analysis of lung cancer; however, the data that they are collecting on what type of treatments the patients received or how they responded to those treatments are scanty. There is a NCIC trial of adjuvant therapy in which 50% of patients receive adjuvant cisplatin-vinorelbine and this trial has been able to accrue patients willing to provide tissue. To date, they have tissue from a few hundred patients with the goal of finding markers predictive of sensitivity. Further, these data are being collected in an adjuvant setting so results will not be linked to initial response - just time to recurrence and death. It should be noted that we have also searched bioinformatic databases, such as that held by the European Bioinformatics Institute (EBI,http://www.ebi.ac.uk/Information) which includes data on PAPSS1 expression obtained from 340 experiments representing 95 different disease states. These data would suggest that PAPSS1 is not differentially expressed in lung cancer cell lines. We have also searched the cBioPortal database which indicates that 6.9% of breast cancer patient xenografts have PAPSS1 amplification and about 2% of lung adenocarcinomas have PAPSS1 mutations. None of these existing datasets, however, link PAPSS1 expression levels to treatment responses to cisplatin.

In terms of our screen results, it is worth noting that several other siRNA screens have been conducted to identify cisplatin modulators in human cell lines, but none of these focused on lung cancer cells exposed to low-dose cisplatin. Supplementary Table S4 displays our screen results for 30 genes that were previously identified by other groups as cisplatin modulators. Swanton *et al*. performed an RNAi screen with 779 kinases to identify modulators of paclitaxel and cisplatin in A549 and two other non-NSCLC cell lines [21]. Even though our experimental conditions, endpoint assay, and method of data analysis differ, our results have considerable overlap with those described by Swanton and colleagues. For instance, *STK16*, a serine/threonine protein kinase that sensitized the cell lines to paclitaxel and cisplatin treatment in the Swanton *et al*. study [21], was also identified as one of the top cisplatin enhancers in our screen (Table S1 and Table S4). Similarly, *CDK5R1* silencing potentiated cisplatin activity in our screen as well as that performed by Swanton *et al*. (Table S4). In our screen, *CDK5R1* silencing was associated with a 23% loss in cell viability in the absence of cisplatin and a further 40% reduction in cell count in the presence of cisplatin. Although considered as a “hit”, the loss of cell viability with *CDK5R1* knockdown alone would lower its ranking in our screen. It should be noted that PAPSS1 was not included in the screen completed by Swanton *et al*.

RNAi screens for cisplatin modulators were also performed in cervical malignant HeLa cells, SKOV3 ovarian carcinoma cells, and BJET-p53KD immortalized human fibroblast cells [17, 22, 23]. Bartz *et al*. identified multiple genes involved in DNA damage repair (*BRCA1*, *BRCA2*, and *REV3L*) as cisplatin potentiators in HeLa cells which were further supported by similar results obtained by Nijwening *et al*. in fibroblast cells. These same genes also significantly enhanced cisplatin activity in our screens but when these genes were silenced in the absence of cisplatin, approximately 30% reduction in cell viability was observed. For this reason, these “hits” were not included in our initial data assessment. It is worth noting that one of the top protein kinases identified in our screen was *RPS6KA3* (Table S1), which codes for RSK2, a protein kinase involved in cell cycle progression when the Ras-ERK pathway is activated. This particular target has recently been validated as a cisplatin activity enhancer in ovarian cancer cells [24]. Our rich dataset along with our stringent target selection approach therefore provide comparable results to previously published screens while also uncovering previously unrecognized cisplatin enhancers such as PAPSS1.

Given the biological role of PAPSS1 [11, 25–27], one can speculate on the role of sulfur metabolism and homeostasis in cancer cells when they are first exposed to cytotoxic agents such as cisplatin. Previous studies have shown that low methionine diets could reduce the concentration of inorganic sulfate in the serum of rats and the hepatic concentration of PAPS [28]. It is also known that many cancer cells, but not normal cells, are methionine-dependent [29, 30]. However, our analysis of yeast deleted for the genes encoding ATP sulfurylase and APS kinase reveal that neither activity in amino acid metabolism is important for cisplatin sensitivity (Figure S5). These results support a model in which PAPSS1 knockdown-induced sensitization to DNA damaging agents in cancer cell lines is related to an evolutionarily specialized role of sulfation, rather than the conserved role in amino acid metabolism that is shared with yeasts. Furthermore, both MET3 and MET14 localize to the cytoplasm of yeasts [31], consistent with the distribution of PAPSS2 in humans [26]; whereas, human PAPSS1 localizes to the nucleus, suggesting that sulfation plays important roles within the nucleus of human cells that might be responsible for the phenotype being observed in our studies. Based on the results presented here, sulfation reactions may play a more important role in the survival of the cancer cell population than previously recognized. PAPS is the required source of biological sulfate for all sulfotransferases [32] which are involved in second phase metabolism of xenobiotics. Therefore, one could postulate that PAPSS1 inhibition may affect the availability of thiol-containing compounds and the metabolism of cisplatin and other anticancer agents [8, 32, 33]. It is known that, mutations in sulfotransferase 1A1 (SULT1A1) are associated with increased lung cancer risk, especially for cigarette smokers [34]. Silencing of SULT1A1 and several other sulfotransferases also appear to enhance cisplatin activity in our WGS (data not shown), albeit not to the same extent seen when PAPSS1 was silenced. Although sulfation likely plays a role in cisplatin metabolism, the effects of PAPSS1 silencing on increasing the activity of radiation, mitomycin C and topoisomerase I inhibitors cannot be explained by the role of sulfotransferases in drug metabolism.

Thus far, our yeast data and the broad-spectrum sensitization to DNA-targeting chemotherapeutics and radiation observed have ruled out the two mechanisms of action: sulfate usage for amino acid metabolism and detoxification of xenobiotics. Our third speculated mechanism of action involves post-translational sulfation of proteins. However, with very little known about PAPSS1 in the literature and the numerous roles that sulfation plays in cell biology, identification of the sulfated protein(s) responsible for the observed sensitizations is beyond the scope of this study. The exact mechanism by which PAPSS1 enhances the activity of multiple cytotoxic agents will take time to fully elucidate, particularly since the cellular responses to genotoxic agents, cisplatin for example, are still not completely understood even though these agents continue to form the mainstay of chemotherapy for patients with cancer [35]. We believe that the flow cytometric and colony formation assay data suggest that the lack of PAPSS1 function alone can induce apoptosis, leading to accumulation of some DNA damage as detected by γH2AX staining, reducing the survival of A549 cells. These data actually suggest that the effects achieved when cisplatin is combined with PAPSS1 silencing are highly synergistic [36]. Consistent with our results (Figure 6), cells without PAPSS1 suppression arrest at the G2/M transition when treated with cisplatin [37] and may remain in this phase for days before committing to apoptosis or surviving by progressing through the cell cycle [37]. However, instead of seeing a further increase in the accumulation of cells at the G2/M transition, cells lacking PAPSS1 expression in the presence of cisplatin accumulated in the G1/S phase. This was associated with upregulation of cyclin E which controls the G1/S transition and downregulation of cyclin A, which is responsible for S/G2 progression [38, 39]. It could be speculated that the cells are progressing to G1/S phase to initiate DNA replication but fail to progress further into late S and G2/M phases of the cell cycle [39]. The amount of DNA damage induced by cisplatin and topotecan was quantified by measuring γH2AX (Figure 7), which is formed in the presence of DNA strand breaks to recruit DNA repair proteins [13]. With cisplatin and topotecan, more DNA damage was detected when cells had reduced PAPSS1 expression. More importantly, there is significantly more cells with multiple γH2AX foci in PAPSS1-silenced cells at all doses and it is reasonable to assume that cells with multiple γH2AX foci are more likely to commit to cell death. Altogether, these results suggest greater accumulation of DNA damage when PAPSS1 expression is low and this could be due to either increased DNA damage or reduced rates of DNA repair. Whether the reduction in PAPSS1-mediated sulfation is indirectly responsible for impairment of DNA repair mechanisms or is an autonomous mechanism contributing to the synergy with cisplatin remains to be established.

Although PAPSS1 silencing sensitized A549 cells to platinum-based agents as well as the non-platinum DNA crosslinker mitomycin C, topoisomerase I inhibitors, and radiation, silencing PAPSS1 did not sensitize A549 cells to topoisomerase II inhibitors. A recent publication by Maede *et al*. demonstrates that topoisomerase I and topoisomerase II inhibitors induce different types of DNA lesions, which are in turn repaired by different pathways [40]. Similarly, Cummings *et al*. reported that suppression of ERCC1, a gene involved in both nucleotide excision repair (NER) and homologous recombination repair, sensitizes prostate cancer cells to both mitomycin C and cisplatin while inhibition of XPA, which is only involved in NER, only sensitizes the cells to cisplatin [25]. The results we presented here suggest that PAPSS1 silencing may be associated with impairment of particular DNA repair mechanism(s) that consequently sensitize cancer cells to specific anticancer agents depending on the DNA lesions induced and the cellular processes that are important for repairing those lesions.

The data reported in Figure 3 and 4 suggest that strong PAPSS1 inhibition enhances cisplatin activity in four different NSCLC cell lines and these data stress the importance of identifying small molecule inhibitors of PAPSS1 as siRNA therapeutic approaches will likely be difficult to deliver in therapeutically relevant doses to achieve sufficient inhibition of PAPSS1 within a heterogeneous population of tumor cells. In this regard, we tested chlorate, a PAPSS inhibitor that inhibits the first step of PAPS synthesis by blocking ATP sulfurylase activity [41]. When cells were pretreated with a non-toxic dose of chlorate (50 mM), the cisplatin IC_50_ reduced by about 2-fold (Figure S6). Chlorate has been used by multiple groups to inhibit sulfation [41, 42] and previous reports have shown that chlorate can halt cell cycle progression at the S phase [43], a result similar to that observed in our study in PAPSS1-silenced cells treated with cisplatin (Figure 6). We have not pursued these studies further because chlorate is a non-isoform-specific inhibitor that is active only when added at very high concentrations. This agent cannot be pursued as a therapeutic compound against PAPSS1 and it is not appropriate as a tool compound to help us understand the mechanisms through which PAPSS1 inhibition causes increased sensitivity to DNA damaging agents. Moving forward, a small molecular inhibitor screen would be a rational approach to identify and validate potent PAPSS1 inhibitors for use as a therapeutic agent. This interaction between PAPSS1 silencing and enhancing the activity of several distinct classes of commonly used anticancer agents is unique and is worth pursuing therapeutically in cancers where these drugs are commonly used.

## EXPERIMENTAL PROCEDURES

### Cell culture and reagents

The NSCLC cell lines A549, H460, H1703, and H358 were obtained directly from Dr. John Minna's laboratory (Dallas, TX) and maintained at 37°C and 5% CO_2_ in RPMI 1640 (Gibco) supplemented with 2 mM L-glutamine (Gibco) and 10% fetal bovine serum (Gibco). Human Bronchial Epithelial Cells (Cell Applications) were maintained in Bronchial/Tracheal Epithelial Growth Medium (Cell Applications) at 37°C and 5% CO_2_. HBEpC cells beyond passage 3 were not used for experiments. PAPSS1 (1:1000) primary antibody was obtained from Abcam while β-Actin (1:50000), cleaved caspase-3 (1:1000), Cyclin A2 (1:2000), Cyclin E1 (1:1000), and cleaved PARP (1:1000) primary antibodies were purchased from Cell Signaling Technology. The chemotherapeutics cisplatin, carboplatin, irinotecan, topotecan, and docetaxel were obtained as ready-to-inject solutions from Hospira. Epirubicin and doxorubicin were obtained from Pfizer while oxaliplatin and mitomycin C were purchased from Sanofi Aventis and Novopharm respectively.

### siRNA Kinome and genome screens

A549 cells were seeded at 2000 cells/well in 384-well plates (Greiner Bio-One). Cells were transfected with the human siGENOME library (Dharmacon) with each well containing a SMARTpool of 4 siRNA duplexes targeting one of the 21, 121 genes within the human genome. Transfection was performed 24 hours after plating using the lipid reagent RNAiMAX (Life Technologies). To ensure minimal toxicity from the transfection reagent and maximal gene silencing, the amount of RNAiMAX that caused less than 10% cell death when complexed with a non-targeting siRNA (47 μL per nmol of siRNA) and the siRNA concentration (25 nM) that consistently caused the most A549 cell death with polo-like kinase 1 (PLK1) knockdown, were selected for the screen. At 24 hours post-transfection, cisplatin was added to achieve a final concentration of either 0 or 0.551 μM (the IC_10_ against A549 cells under the screening conditions) in triplicates. The cells were fixed with 95% ethanol and the nuclei were stained with 16.2 μM of Hoechst 33342 (Life Technologies) at 72 hours following cisplatin treatment. The plates were then imaged using the IN Cell Analyzer 1000 (GE Healthcare), an automated fluorescent microscopy platform that enables high content screening. Cell counts were determined via the IN Cell Developer Toolbox software.

To validate the screening conditions, a preliminary kinome screen (PKS) was performed with a subset of the siGENOME library targeting 640 protein kinases using the protocol described above. The whole genome screen (WGS) was subsequently completed. Each plate had four controls: RNAiMAX lipid control, PLK1, scramble (non-targeting siRNA), and BRCA2 (siRNA targeting breast cancer type 2 susceptibility protein). PLK1 served as a transfection efficiency control [44] while BRCA2, the silencing of which is a known cisplatin potentiator, was a positive control [17]. These controls were randomly spotted in the first four columns of each plate to account for positioning effects. For quality control, full plates of controls with no siRNA, +/− lipid, and +/− cisplatin were screened in triplicates once a week. A cisplatin dose response curve was also generated on each cisplatin treatment day of the screen to ensure accurate drug dilution and addition.

Nine images were taken per well and the median cell counts were used to compare cell survival in untreated versus cisplatin-treated conditions when individual genes were silenced. A “survival index” was calculated to determine the lethality of gene knockdown: the cell number was normalized such that 100% would be equivalent to the median cell count obtained from lipid controls within the same plate. The difference in cell number in untreated versus cisplatin-treated cells indicates the extent of cisplatin potentiation. This value for each gene was normalized to that obtained from BRCA2-silencing and multiplied with the survival index to achieve a “gene score”, which was used to rank all the genes.

The screen results were validated through three independent experiments where the top 20 kinases from the PKS were targeted with a pool of three different Stealth siRNA duplexes (Life Technologies) using the same methodologies as the screens. Additional validation work was performed by generating cisplatin dose response curves with the top five targets: cells were stained with Hoechst for total nuclei count and ethidium homodimer I (Biotium) for dead cells at 72 hours following cisplatin treatment and imaged with the IN Cell Analyzer after a 20-minute incubation at 37°C. Cells were classified as “dead” if they showed > 30% overlap of the two stains. A linear mixed effects model was applied to the siRNA screen to account for differences in cell counts due to well location, pipetting, and plate-to-plate variations. The Benjamini-Hochberg method was used for multiple test comparisons and all hits identified in this study were statistically significant based on their adjusted *p*-values.

### siRNA transfection for PAPSS1 validation studies using multiple NSCLC cell lines

The indicated NSCLC cells were seeded manually in 384-well plates at 50 μL/well of OPTI-MEM reduced serum media (Life Technologies) supplemented with 4% FBS and 2 mM L-glutamine. Briefly, RNAiMAX was diluted in OPTI-MEM and complexed with a pool of three different Stealth siRNA sequences targeting PAPSS1 (HSS113394, HSS189820, HSS189821; Life Technologies) for 20 minutes and then added to the cells. The Stealth RNAi negative control kit (Life Technologies) was used as scramble control.

### RNA extraction and qRT-PCR

Cells were seeded in 6-well plates and transfected with the indicated siRNAs as described above. Total RNA was extracted 48 hours post-transfection using the RNeasy Mini Kit (Qiagen) and quantified using the ND-1000 Spectrophotometer (NanoDrop Technologies). The QuantiTect Reverse Transcription Kit (Qiagen) was used to eliminate genomic DNA and to synthesize cDNA from 1 μg of total RNA. Quantitative Real-time PCR was performed in triplicates via the 7900HT system (Applied Biosystems) using 2X TaqMan fast advanced master mix and 20X Taqman PAPSS1 (Hs00968937_m1) and GAPDH (hCG2005673) probes (Applied Biosystems) as per manufacturer's instructions. Data were analyzed using the SDS 2.2 software (Applied Biosystems) and the relative messenger RNA quantity was calculated using the ddCt method with GAPDH as the endogenous control.

### SDS-PAGE and western blot analysis

All buffer chemicals were obtained from Sigma-Aldrich. Cells were cultured in 6-well plates and transfected with siRNA the following day. At 72 hours post-transfection, cells were lysed with buffer containing 50 mM Tris-HCl (pH 7.4), 150 mM NaCl, 0.25% sodium deoxycholate, 1% NP-40, 0.1% SDS, 1 mM EDTA and Mini Protease Inhibitor Cocktail tablets (Roche Diagnostics). Cellular lysates were clarified (20 min at 14000g) and protein concentrations were determined using the BCA Protein Assay Kit (Pierce). Lysates (30 μg) were separated by SDS-PAGE on a 4–12% Bis-Tris gel (Life Technologies) and transferred to 0.2 μm nitrocellulose membranes using the Trans-Blot Turbo Transfer System (Bio-Rad Laboratories). The membranes were blocked for 1 hour (5% skim milk in TBST [Tris buffered saline + Tween: 20 mM Tris-base, 140 mM NaCl, 0.1% Tween 20]) and incubated in blocking solution with primary antibodies (1:1000) overnight at 4°C. The membranes were washed with TBST (3 × 5 min) and then incubated with horseradish peroxidase-conjugated secondary antibodies (Promega) at room temperature for 1 hour (1:10000 for β-actin and 1:5000 for all others). The blots were then washed with TBST as before, developed using the Super Signal West Pico Chemiluminescent Substrate (Thermo Scientific), and visualized using the ChemiDoc MP System and the ImageLab imaging software (Bio-Rad). Protein quantification was performed using the ImageLab software.

### Flow cytometry

Cells were plated and transfected in 6-well plates at 0.094 fmol siRNA per cell. At 24 hours post-transfection, cells were treated with the indicated doses of cisplatin. At the indicated time points, the supernatant and adherent cells harvested by trypsinization were collected. For cell cycle analysis, the cells were washed with HBSS and fixed in 70% ethanol at 10^6^ cells/mL for 1 hour on ice followed by an overnight incubation at −20°C. Cell pellets were collected by centrifugation and then stained in PBS buffer containing 50 μg/mL propidium iodide (Invitrogen), 1 mg/mL RNase A (Sigma), and 0.1% Triton X-100 (Bio-Rad) for 15 min at 37°C followed by 1 hour on ice. To assess DNA damage, transfected cells were treated with cisplatin or topotecan for 48 hours, and then fixed and stained with anti-γH2AX antibody and counter-stained with Sytox^®^green nucleic acid stain according to manufacturer's instructions included in the Apoptosis, DNA damage, and Cell Proliferation Kit (BD Pharmingen). A 24-hour treatment with 250 nM of topotecan was used as a positive control. Data were acquired and analyzed using the FACSCalibur flow cytometer and the WinMDI 2.9 software respectively. Based on sytox^®^green fluorescent intensity, cells with less than 2N DNA were excluded from analysis of γH2AX expression.

### Immunofluorescence staining and high content analysis

Cells were seeded in a 96-well plate and transfected as described above. Cells were treated with different concentrations of cisplatin or topotecan for 48 hours, after which the cells were fixed and permeabilized using the BD Cytofix/Cytoperm^TM^ Fixation/Permeabilization Kit (BD Pharmingen) as per manufacturer's instructions. Non-specific binding was blocked by incubating fixed cells with 100 μL of BD Pharmingen^TM^ Stain Buffer per well for 30 minutes at room temperature. H2AX phosphorylation was labeled using the Alexa Fluor 647 Mouse anti-γH2AX (Ser139) antibody (BD Pharmingen) as per manufacturer's instructions and the nuclei were counter-stained with 2 μg/mL of Hoechst 33342. Nine imaging fields/well were acquired using the IN Cell Analyzer 2200 (GE Healthcare) equipped with 20x objective and the data were analyzed using the IN Cell Analyzer Workstation 3.7 software (GE Healthcare).

### Colony-formation assay

A549 cells were transfected in 6-well plates. After 24 hours, the cells were exposed to an empirically determined IC_10_ (0.71 μM) of cisplatin for 24 hours, harvested by trypsinization, and then seeded at 500 cells per well in triplicates. The cells were then incubated at 37°C, 5% CO_2_ for 14 days without disturbance. The colonies formed were fixed with 6.25% glutaraldehyde (Sigma) and stained with 0.5% crystal violet (Sigma) for 30 minutes, washed with distilled water, dried overnight, and counted the next day. Plating efficiency (PE) was defined as the percentage of trypan blue excluding cells that formed colonies of > 50 cells (*PE* = [(no. of colonies formed/no. of cells seeded) x100%]). For radiation studies, cells were transfected for 48 hours and then irradiated at 10^6^ cells/mL at the indicated doses. Cells were then plated in triplicates to achieve 100–500 colonies 12 days later. Cells were stained with aqueous malachite green and dried overnight prior to colony counting. The surviving fraction (SF) was calculated using the equation *SF* = [no. of colonies formed after treatment/ (no. of cells seeded x PE)].

### Dose response curves

With the exception of the radiation study (see colony-formation assay methods), all dose response curves were conducted in 384-well plates. Cells were exposed to various concentrations of selected agents 24 hours following transfection. Cell viability was determined 72 hours post-treatment using the IN Cell Analyzer as described above.

### Statistical analysis

Dose response curves were plotted using Prism 6.0 (GraphPad Software) as mean ± SEM from at least three independent experiments. IC_50_ values were interpolated from the fitted curves and compared for statistical differences using one-way ANOVA followed by Tukey adjustments (top five gene targets) or the Student's *t*-test (scramble vs. PAPSS1). Student's *t*-test was used to determine the statistical significance of the differential PAPSS1/GAPDH mRNA expressions and the differences in IC_50_ values of various chemotherapeutics between Scramble and PAPSS1 siRNA-transfected cells. The radiation data were fitted in the linear-quadratic model in SPSS 22. A *p*-value of < 0.05 was considered statistically significant.

## SUPPLEMENTARY FIGURES AND TABLES


