# Supplementary material for: 3′-Phosphoadenosine 5′-phosphosulfate synthase 1 (PAPSS1) knockdown sensitizes non-small cell lung cancer cells to DNA damaging agents
Source: Oncotarget. 2015 Apr 11;6(19):17161–77. doi: 10.18632/oncotarget.3635 (PMC4627299; doi:10.18632/oncotarget.3635)
Supplement: Supplementary file 1 [file oncotarget-06-17161-s001.pdf]

SUPPLEMENTARY EXPERIMENTAL PROCEDURES

Yeast strains and methods

The haploid *met3Δmet14Δ* double mutant (MATa *ura3Δ0 his3Δ1 met15Δ0 leu2Δ0 lys2Δ0 met3Δ*::*KanMX met14Δ*::*KanMX*) was generated in this study from the individual heterozygous diploid strains *met3Δ*::*KanMX*/*MET3* and *met14Δ*::*KanMX*/*MET14* [1]. Other haploid deletion strains were obtained from the MATa yeast knockout collection (Open Biosystems). Yeast strains were grown in YPD or synthetic dextrose (SD) medium containing only amino acids essential to complement the auxotrophies present in the strains.

Spot dilution assays [2] and cisplatin cytotoxicity assays [3] were performed as described using the WT strain BY4741 as control. Cytotoxicity was assessed using a colony formation assay by plating cell dilutions on nonselective YPD agar plates following a 4 hour treatment of 6×10^6 cells with the indicated cisplatin concentration (0, 0.125, 0.25, 0.5, and 1.0 mM). The number of colonies was counted after 2 days of growth at 30°C.

SUPPLEMENTARY REFERENCES

1. Pan X, Yuan DS, Xiang D, Wang X, Sookhai-Mahadeo S, Bader JS, Hieter P, Spencer F, Boeke JD. A robust toolkit for functional profiling of the yeast genome. Molecular cell. 2004; 16:487–496.
2. Huang RY, Eddy M, Vujcic M, Kowalski D. Genome-wide screen identifies genes whose inactivation confer resistance to cisplatin in Saccharomyces cerevisiae. Cancer resea rch. 2005; 65:5890–5897.
3. Rodriguez-Lombardero S, Vizoso-Vazquez A, Lombardia LJ, Becerra M, Gonzalez-Siso MI, Cerdan ME. Sky1 regulates the expression of sulfur metabolism genes in response to cisplatin. Microbiology. 2014; 160:1357–1368.

SUPPLEMENTARY FIGURES AND TABLES

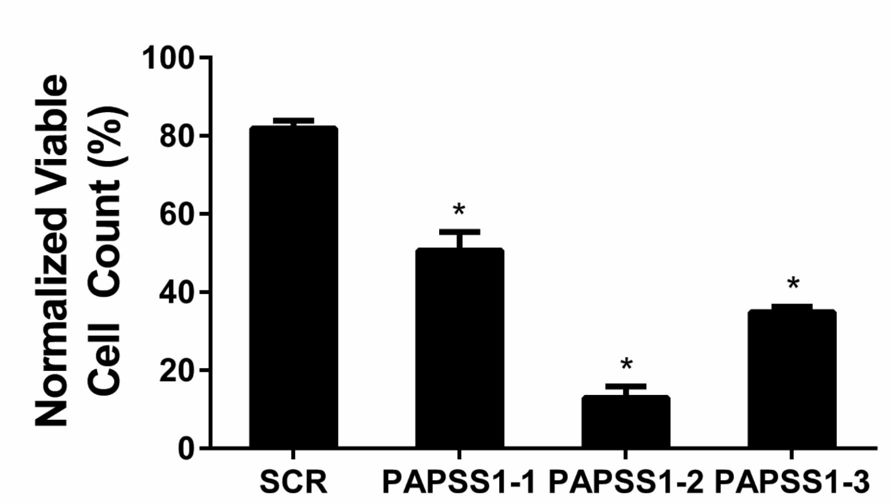

Supplementary Figure S1: Sensitization of A549 cells to cisplatin treatment is observed with three different PAPSS1-targeting siRNAs. A549 cells transfected with 25 nM of either non-targeting (scramble; SCR) or PAPSS1-targeting siRNA were treated with low-dose cisplatin. The viable cell count at 72 hours following drug treatment is normalized such that 100% is equivalent to the cell viability of scramble-transfected, untreated controls. The three siRNA sequences used here are 1) 5'- GCAAATTCATGAAGGTGCAAGTTTA-3', 2) 5'- GATGCTGGCTTAGTGTGCATCACAA-3', and 3) 5'-GGGAGTACTTGCAGTGCCTTCATTT-3', targeting exons 4, 3, and 7 respectively. These three siRNA duplexes were pooled for the remaining validation studies to minimize off-target effects without compromising on-target knockdown. Statistical analyses were performed using one-way ANOVA followed by Tukey adjustment for multiple test comparisons (data plotted as mean ± SD; *$p < 0.05$).

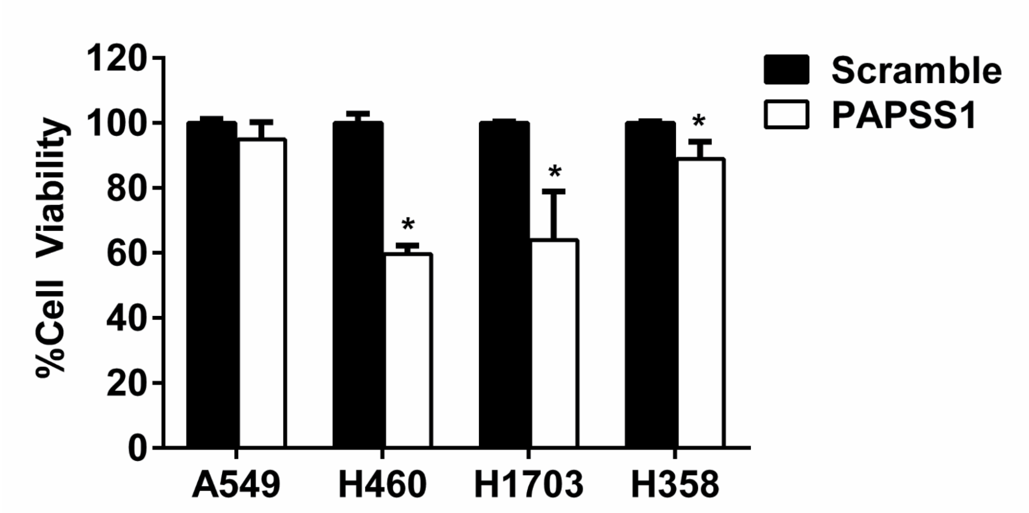

Supplementary Figure S2: PAPSS1 knockdown causes variable loss in cell viability in different cell lines. Depending on the cell line, loss of PAPSS1 expression could result in cell death (data plotted as mean ± SEM; *$p < 0.05$).

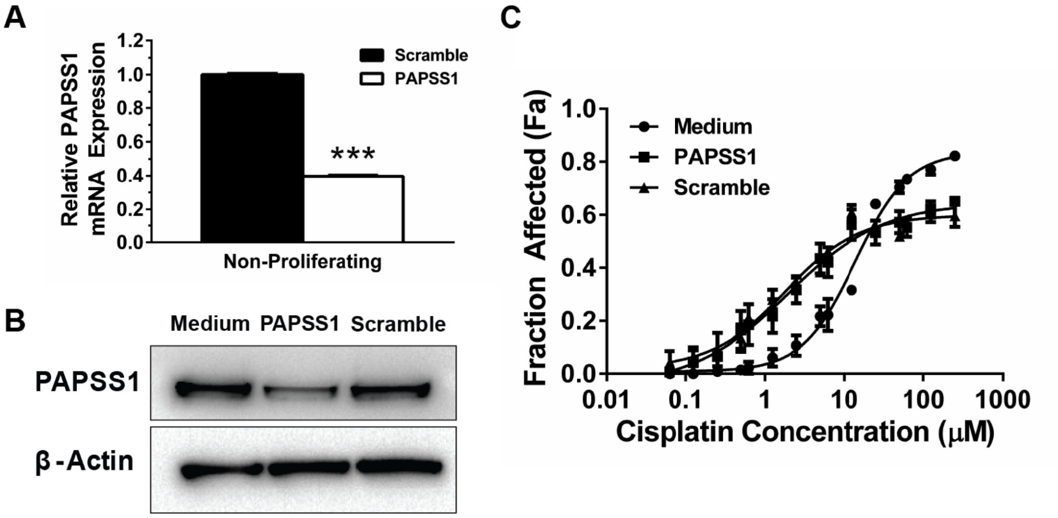

Supplementary Figure S3: Substantial knockdown could not be achieved in HLMVEC and no change in the cisplatin dose response was observed. Despite using the highest non-toxic dose of lipid-siRNA complex, the reduction of PAPSS1 mRNA levels was less than 70% **A.** and changes in protein expression in the presence of the siRNA were minimal **B.**. Under these conditions, sensitivity to cisplatin did not differ between PAPSS1 and Scramble controls (**C.**; data plotted as mean ± SEM). The sensitization observed compared to medium control could be attributed to lipid toxicity from the transfection.

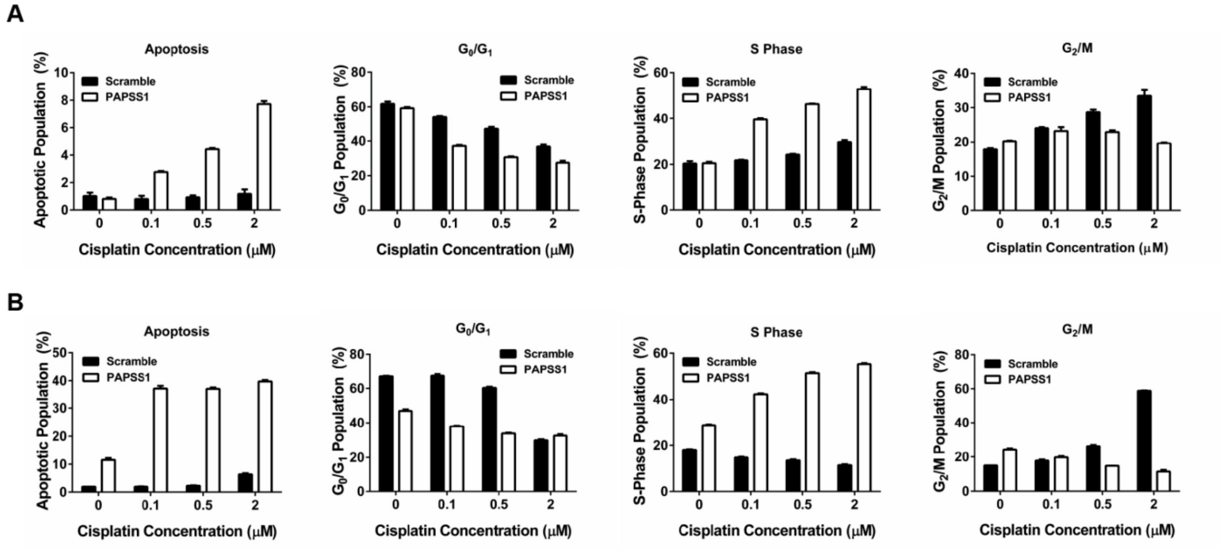

Supplementary Figure S4: PAPSS1 silencing induces apoptosis and causes A549 cells to accumulate in the S phase in the presence of cisplatin. At 24 **A.** and 48 **B.** hours following cisplatin treatment, cells transfected with scramble siRNA arrest at the G2/M phase in a dose-dependent manner. Cells with reduced PAPSS1 expression are much more apoptotic relative to scramble controls and tend to accumulate at the G1/S phase. Data are plotted as mean ± SD from three replicates.

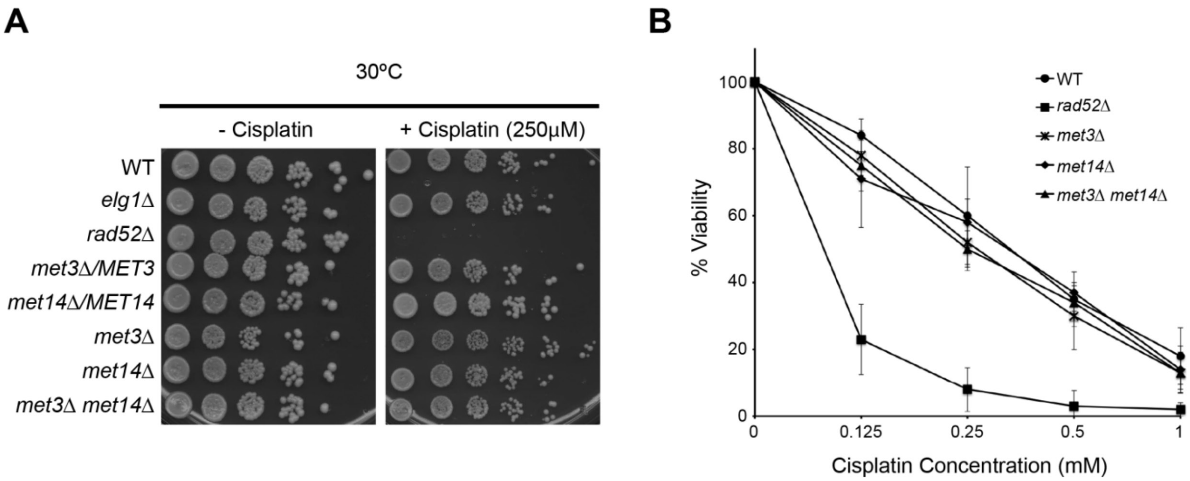

Supplementary Figure S5: Yeast lacking PAPS synthase activity is not sensitized to cisplatin. **A.** Ten-fold spot dilution assays of the indicated strains grown on SD medium with or without cisplatin (250 µM) for 2 days at 30°C. The *met3Δmet14Δ* double mutant lacks both enzymatic components of yeast PAPSS, *elg1Δ* and *rad52Δ* serve as weak and strong cisplatin-sensitive controls respectively, and are compared to an isogenic wildtype (WT) strain. **B.** The cisplatin cytotoxicity of *met3Δmet14Δ* double mutants was comparable to the WT control. The percentage viability of the cells after a pulse of high dose cisplatin was quantified, and normalized to untreated controls (mean ± SD; $n = 3$).

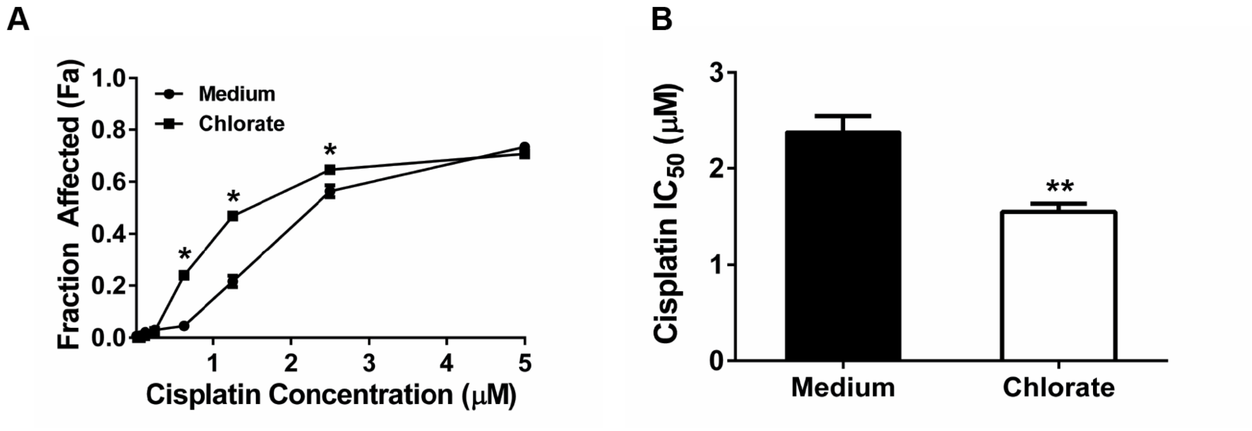

Supplementary Figure S6: Pre-treatment with chlorate (50mM) causes ~2-fold leftward shift in the cisplatin dose response curve. A549 cells were pre-treated with medium or 50 mM sodium chlorate for 24 hours prior to cisplatin exposure for 72 hours. Data are plotted as mean ± SEM ($n = 4$). Individual doses were compared for statistical significance using the Student's *t*-test (*$p < 0.05$). Cells treated with sodium chlorate had a cisplatin IC_{50} of about 1.2 µM, which is almost two-fold lower than that of the medium control (2.1 µM) (B; mean ± SEM; **$p < 0.01$).

Supplementary Table S1: Top 20 Kinases from siRNA Screens

Kinase Rank	PKS Kinase	WGS Kinase
1	PIP5K2A	PIP5K2A
2	CDC42BPA	STK16
3	PRKWNK4	SPEC2
4	LTK	PIK3R1
5	BLK	RPS6KA3
6	FN3K	PTK9L
7	PAPSS1	PRKAA2
8	MAP3K14	ALS2CR2
9	ALS2CR2	FLJ35107
10	FASTK	SIK2
11	ILKAP	PKIB
12	PTPRG	PRKWNK4
13	MAP3K3	FASTK
14	PTK9L	RPS6KA6
15	DKFZP586B1621	DKFZP586B1621
16	CDC7	DUSP10
17	MAP4K2	BLK
18	PRPS2	PAPSS1
19	PCTK2	STK32A
20	PKIB	PANK3

The top 20 kinases identified from the preliminary kinase screen (PKS) and the whole genome screen (WGS) are listed here. These kinases were identified based on their Gene Score, which is a calculated value based on viable cell count from gene knockdown alone and the differences in cell count with gene knockdown in the presence versus the absence of low-dose cisplatin.

Supplementary Table S2: Top 10 Kinases from siRNA Validation Screens

Rank	Validation 1	Validation 2	Validation 3
1	PAPSS1	PAPSS1	PAPSS1
2	PIP5K2A	PIP5K2A	PIP5K2A
3	TWF2	ILK	TWF2
4	PIK3C2A	NEK1	PRKWNK4
5	PRKWNK4	TWF2	ILK
6	NEK1	PRKWNK4	ALS2CR2
7	PAG	ILKAP	PIK3C2A
8	MAP3K3	PIK3C2A	NEK1
9	LTK	BLK	ILKAP
10	ILK	PAG1	MAP3K14

The top kinases identified from the kinome and genome screens were validated in three independent experiments using the same protocol but different siRNA sequences. The table lists the top 10 kinases from the three experiments based on gene score (see methods for siRNA screens).

Supplementary Table S3: NSCLC Cell Line Characteristics

Cell Line	Tumour Subtype	P53 Status	KRAS Status
A549	Adenocarcinoma	WT	12TGT
H460	Large Cell Carcinoma	WT	61CAT
H1703	Squamous Cell Carcinoma	GAG→AAG	WT
H358	Bronchioalveolar Carcinoma	Homozygous deletion	12TGT

This table details the subtype and the P53 and KRAS mutational status of the NSCLC cell lines used in this study. All four cell lines demonstrated sensitization to cisplatin treatment despite their differing genetic background.

Supplementary Table S4: Comparison fo Hits with Previously Published Screens

		Current Study		References to Published Screens		
	Gene	Viability from Gene Knockdown (%)	Fold-increase in Cisplatin Response	Cisplatin Sensitizer	Lethal Gene	Non-Sensitizer
1	ATR	100	1.9	[1, 2, 3]		
2	BARD1	70	3.6	[2]		
3	BRCA1	75	4.2	[2, 3]		
4	BRCA2	73	5.0	[2, 3]		
5	BRIP1	98	2.0	[2]		[3]
6	CALM1	94	4.0	[1]		
7	CDK5R1	77	4.3	[4]		
8	CERT	73	2.2	[4]		
9	CHEK1	32	0.3	[1, 2]	[3]	
10	EPHB1	93	2.9	[1]		
11	GSK3B	90	2.2	[1]		
12	MAD2L2	77	3.9	[2]		
13	MAP4K2	94	3.1	[1]		
14	MARCKS	87	4.9	[1]		
15	NRGN	106	4.3	[1]		
16	PKIA	85	3.1	[1]		
17	PRKAB1	47	1.0	[1, 2]		
18	PRKCN	75	3.8	[1]		
19	PSKH2	78	2.7	[4]		
20	PTK9L	91	6.0	[1]		
21	RAD18	106	4.5	[2]		
22	RAD51	100	2.3	[2]	[3]	
23	REV1L	89	5.0	[2]		
24	REV3L	70	4.5	[2, 3]		
25	RFWD3	89	4.9	[2]		
26	SHFM1	67	3.9	[2]	[3]	
27	STK16	102	6.2	[4]		
28	STK22D	95	3.2	[1]		
29	STK25	90	2.5	[1]		
30	TNFRSF10A	92	3.0	[4]		

[1] Arora S, Bisanz KM, Peralta LA, Basu GD, Choudhary A, Tibes R and Azorsa DO. RNAi screening of the kinome identifies modulators of cisplatin response in ovarian cancer cells. Gynecol Oncol. 2010.

[2] Bartz SR, Zhang Z, Burchard J, Imakura M, Martin M, Palmieri A, Needham R, Guo J, Gordon M, Chung N, Warrener P, Jackson AL, Carleton M, Oatley M, Locco L, Santini F, et al. Small interfering RNA screens reveal enhanced cisplatin cytotoxicity in tumor cells having both BRCA network and TP53 disruptions. Mol Cell Biol. 2006; 26(24):9377–9386.

[3] Nijwening JH, Kuiken HJ and Beijersbergen RL. Screening for modulators of cisplatin sensitivity: unbiased screens reveal common themes. Cell Cycle. 2011; 10(3):380–386.

[4] Swanton C, Marani M, Pardo O, Warne PH, Kelly G, Sahai E, Elustondo F, Chang J, Temple J, Ahmed AA, Brenton JD, Downward J and Nicke B. Regulators of mitotic arrest and ceramide metabolism are determinants of sensitivity to paclitaxel and other chemotherapeutic drugs. Cancer Cell. 2007; 11(6):498–512.

Results from thirty genes in the siRNA screen presented in our study are compared with that from four different screens conducted in human cells for cisplatin modulators by other groups.
